# The Personal Human Oral Microbiome Obscures the Effects of Treatment on Periodontal Disease

**DOI:** 10.1371/journal.pone.0086708

**Published:** 2014-01-29

**Authors:** Karen Schwarzberg, Rosalin Le, Balambal Bharti, Suzanne Lindsay, Giorgio Casaburi, Francesco Salvatore, Mohamed H. Saber, Faisal Alonaizan, Jørgen Slots, Roberta A. Gottlieb, J. Gregory Caporaso, Scott T. Kelley

**Affiliations:** 1 Department of Biology, San Diego State University, San Diego, California, United States of America; 2 Graduate School of Public Health, San Diego State University, San Diego, California, United States of America; 3 CEINGE-Biotecnologie Avanzate, Napoli, Italy; 4 Dipartimento di Medicina Molecolare e Biotecnologie Mediche, Università di Napoli Federico II, Napoli, Italy; 5 Section of Endodontics, Herman Ostrow School of Dentistry of USC, Los Angeles, California, United States of America; 6 Professor of Dentistry and Microbiology, Herman Ostrow School of Dentistry of USC, Los Angeles, California, United States of America; 7 BioScience Center, San Diego State University, San Diego, California, United States of America; 8 Department of Biological Sciences, Northern Arizona University, Flagstaff, Arizona, United States of America; 9 Institute for Genomics and Systems Biology, Argonne National Laboratory, Argonne, Illinois, United States of America; Charité-University Medicine Berlin, Germany

## Abstract

Periodontitis is a progressive disease of the periodontium with a complex, polymicrobial etiology. Recent Next-Generation Sequencing (NGS) studies of the microbial diversity associated with periodontitis have revealed strong, community-level differences in bacterial assemblages associated with healthy or diseased periodontal sites. In this study, we used NGS approaches to characterize changes in periodontal pocket bacterial diversity after standard periodontal treatment. Despite consistent changes in the abundance of certain taxa in individuals whose condition improved with treatment, post-treatment samples retained the highest similarity to pre-treatment samples from the same individual. Deeper phylogenetic analysis of periodontal pathogen-containing genera *Prevotella* and *Fusobacterium* found both unexpected diversity and differential treatment response among species. Our results highlight how understanding interpersonal variability among microbiomes is necessary for determining how polymicrobial diseases respond to treatment and disturbance.

## Introduction

Periodontitis is a complex, polymicrobial infection of the periodontium. The disease is caused by dental plaque microorganisms that migrate into the periodontal pocket and give rise to inflammation of the gingiva [1]. Left untreated, the inflammatory process may lead to loss of tooth-supporting connective tissue and bone, and eventually to edentulism [2]. While oral microbes are the principal cause of periodontitis, factors such as tobacco use, osteoporosis, obesity, and diabetes exacerbate the disease [3]. Periodontitis has also been associated with systemic diseases, including atherosclerosis, preterm birth, and diabetes [4].

Conventional diagnostic techniques in periodontics are based on clinical examination and occasionally on laboratory tests. Clinical examination assesses gingival health status, periodontal pocket depth, clinical attachment loss, radiographic alveolar bone level, oral hygiene performance, and other clinical variables [5]. Laboratory testing may include microbiological analysis for periodontal pathogens, blood tests for systemic health status, and histological evaluation of tissue changes. The obtained information allows a classification of periodontal disease into gingivitis and mild, moderate and severe periodontitis. However, the current diagnostic tests are not particularly sensitive and specific for periodontal disease activity and have limited prognosticative value. Rapid molecular techniques capable of identifying periodontal bacteria and viruses with great accuracy may eventually provide a better classification and diagnosis of various types of periodontal disease and aid significantly in clinical decision-making [5].

Thus far, most of what we know about bacteria in periodontal disease has been learned through anaerobic culturing, but the immense bacterial diversity in periodontal pockets will require molecular methods able to simultaneously investigate all members of periodontal pocket communities, including those that we cannot currently grow in culture [6], [7], [8]. Recent studies by Griffen *et al.* (2012) and Abusleme *et al.* (2013) using Next-Generation Sequencing (NGS) of bacterial small-subunit ribosomal RNA (16S rRNA) genes showed the promise of these methods for investigating periodontal disease. [9], [10]. These studies analyzed patterns of microbial diversity in healthy and diseased periodontal pockets and showed clear community level differences among, and even within, individuals.

Here, we used NGS methods to determine how standard periodontal disease treatment, namely scaling and root planing and oral hygiene instruction, altered polymicrobial diversity in periodontal pockets. The study design and analytical methods allowed us to investigate differences in microbial community diversity among periodontal health and disease states, and whether there were consistent associations of particular bacteria with health or disease.

## Materials and Methods

### Ethics Statement

The supporting TREND checklist for this study is available in the supplemental materials (Figure S1). The San Diego State Institutional Review Board obtained full ethical approval on August 11, 2008. Written informed consent was obtained from each participant. The study was registered as “Assess the Effect of Treating Periodontal Disease on Cardiovascular Function in Young Adults” on ClinicalTrials.gov under the identifier NCT01376791.

### Study Population, Clinical Assessment and Treatment

Thirty-six subjects aged 21–40 with gingivitis, mild-to-moderate periodontitis, or severe periodontitis, along with 4 healthy controls were recruited from an American Indian/Alaska Native (AIAN) population in Southern California. The AIAN population is known to have a higher incidence of periodontal disease than the general population, making it an important subject of study for this community [11]. Degree of periodontal disease was assessed by measuring probing pocket depths (PD), clinical attachment loss (CAL), plaque scores, and bleeding on probing (BOP). Twenty-three patients aged 21–40 with gingivitis (CAL≤3 mm, PD≤4 mm, BOP>10%), twelve patients with mild-moderate periodontitis (CAL≥4 mm, PD≥5 mm, BOP≥30%), one patient with severe periodontitis (CAL≥6 mm, pocket depths ≥7 mm, BOP≥30%), along with 4 healthy controls (CAL≤3 mm, PD≤3 mm, BOP≤10%) all aged 21–40 were enrolled in the study. Following completion of periodontal treatment (at least 6 weeks later), patients returned for a follow-up visit.

Patients received a baseline dental examination which included a full dental screening and measurement of periodontal pocket depths of all teeth. Following the clinical examination, microbial samples were collected from the two deepest periodontal pockets of the dentition using a periodontal scaler. The sample material was wiped onto sterile Whatman filters and submerged into 10 mL of sterile Sodium-Magnesium buffer (SM buffer) and kept at 4°C. DNA was extracted with the NucleoSpin Tissue Nucleic Acid and Protein Purification Kit (Macherey-Nagel GmbH & Co, Germany) from the supernatant after vigorous vortexing. The same procedure was repeated at least six weeks following completion of standard periodontal disease treatment. Patients were classified as improved if their average pocket depth decreased (twelve patients), worsened if their average pocket depth increased (eighteen patients), and no change if their average pocket depth remained the same (6 patients) [12], [13].

### Next-Generation Sequencing and Bioinformatics

The 27F and 338R primers targeting the V1–V2 hypervariable regions of 16S rRNA genes were used in the PCR reactions [14]. The primers were barcoded following Fierer *et al.* (2008), using the same PCR thermocycling parameters. PCR products were submitted to the core sequencing facility at the University of Pennsylvania for purification, equimolar dilution and pyrosequencing on a Roche 454 GS FLX instrument. The dataset sequences were deposited into the publicly accessible QIIME Database at http://www.microbio.me/qiime. The study name in QIIME is: Schwarzberg_periodontal_disease. The study ID is: 2083. The sequences were also deposited into figshare at http://dx.doi.org/10.6084/m9.figshare.855613 along with the mapping file at http://dx.doi.org/10.6084/m9.figshare.855612.

Sequencing data were analyzed using QIIME 1.6.0-dev [15]. Briefly, sequences were clustered into 97% using a uclust-based [16] open-reference OTU picking protocol using the Greengenes 12_10 reference sequences [17]. Taxonomy was assigned to sequences using the RDP Classifier [18], retrained on Greengenes 12_10, via QIIME. Representative sequences, which were selected as the centroid sequence of each OTU, were aligned with PyNAST [19], and trees were constructed using FastTree [20] for phylogenetic diversity calculations. Procrustes analysis [21] was performed using QIIME with 1000 Monte Carlo iterations. OTU counts for specific taxonomic groups (e.g., *Streptococcus*) were exported from QIIME for statistical analyses in R version 2.15.1 [22]. Representative *Fusobacterium* and *Prevotella* sequences were exported for multiple sequence alignment and phylogenetic analyses (Figures S3 and S4).

## Results and Discussion

**Figure 1:**
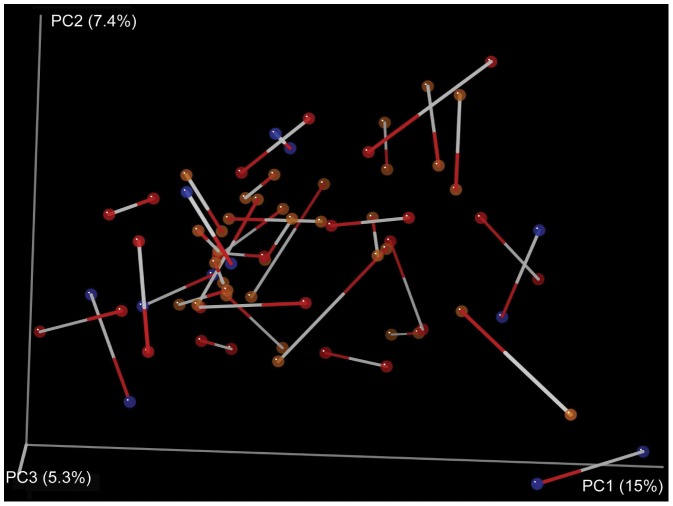
Procrustes analysis of samples before and after periodontal treatment, Procrustes *M^2^* value = 0.420 (dissimilarity of the two datasets), *P*-value = 0.00 based on 1000 Monte Carlo iterations. This analysis is a visualization of a principal coordinates analysis (PCoA) of the Unifrac distances between samples, showing the best superimposition of one Unifrac plot on the other. Samples collected from the same patient before and after treatment are connected by a line, the white end indicating the before-treatment sample red end indicating the after-treatment sample. Patients were classified as improved (red circles), worsened (brown circles) or no change (blue circles). Determination of patient improvement or decline was based on changes in observed pocket depth, a standard approach used in periodontal research [12], [13].

A total of 76 periodontal pocket microbial community samples were analyzed via 454 pyrosequencing of bacterial 16S rRNA amplicons (Figure S2). Pyrosequencing yielded a combined total of 759,717 sequences across all samples with a median sequence count of 9,676. From these data, we identified 87 bacterial genera belonging to 12 different divisions, the majority of which were common members of periodontal pocket microbiota. Community-level analyses (Unifrac-based PCoA) did not uncover clear differences between samples collected prior to treatment with those collected post-treatment, even after accounting for the treatment effectiveness. On the contrary, post-treatment samples remained most similar to pre-treatment samples from the same individual (Figure 1).

**Figure 2:**
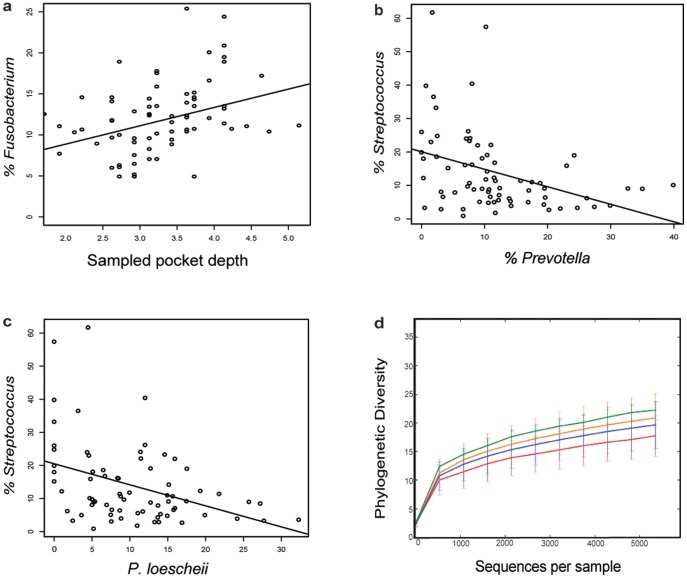
Statistical trends and alpha diversity of samples. **a** Percent of *Fusobacterium* relative to pocket depth of sampled teeth (r = 0.2413, *P* = 0.0411). **b** Percent of *Streptococcus* relative to *Prevotella* (r = −0.3846, *P* = 0.0008). **c** Percent of *Streptococcus* relative to single *Prevotella* species, *P. loescheii* (r = −0.3055, *P* = 0.0090). **d** Rarefaction trends: distribution of number of sequences per sample. Samples were classified as Healthy Controls (red line), gingivitis (blue line), mild/moderate periodontitis (orange line) and severe periodontitis (green line).

Deeper analyses of the distributions of specific bacterial taxa associated with either health (*Streptococcus*, *Veillonella*) or disease (*Fusobacterium*, *Prevotella* and *Leptotrichia*) [6], [9], [10] found only *Fusobacterium* to be significantly correlated with pocket depth over all samples (Figure 2a). As expected, we found an inverse correlation between the abundance of *Fusobacterium* and *Streptococcus* (data not shown) and between *Streptococcus* and *Prevotella* (Figure 2b), with the association primarily driven by the negative correlation between *Streptococcus* and *P. loescheii* (Figure 2c). *Fusobacterium*, especially *F. nucleatum*, plays a key role in periodontal biofilm development by bridging early and late colonizers, according to the successional integration theory [23]. *Streptococcus* species establish the biofilm and *P. loescheii* attaches directly to *Streptococcus*, unlike the other *Prevotella* species. The roles played by these bacterial genera may make them particularly responsive to biofilm disturbance, and perhaps make them useful indicators of periodontal treatment efficacy.

**Figure 3:**
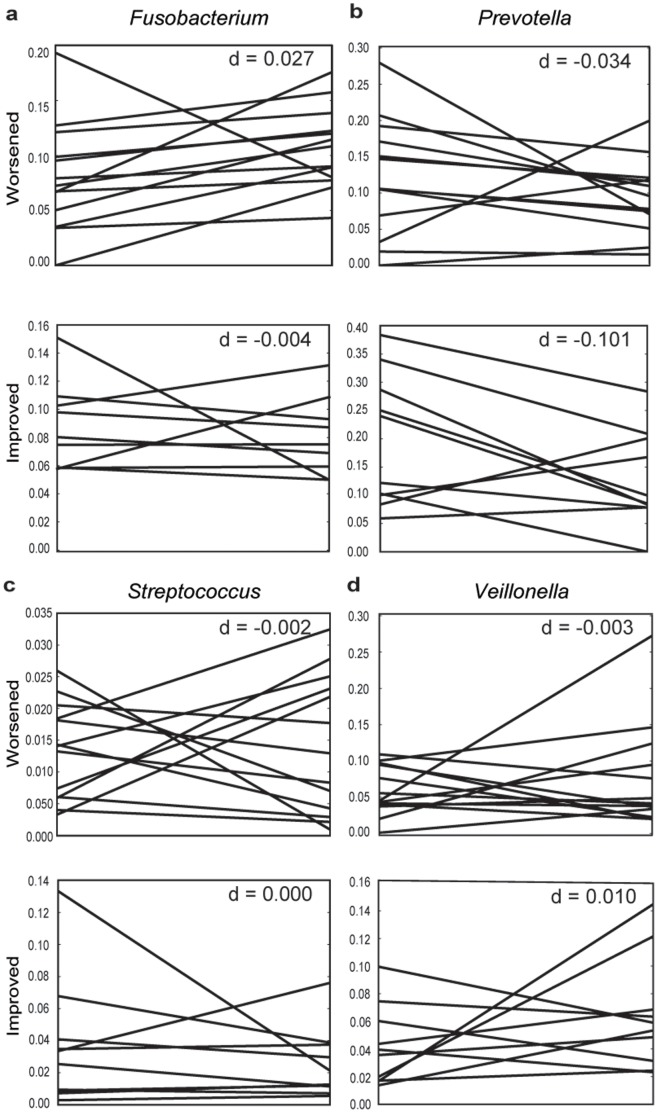
Trends of bacterial genera associated with health or disease, separated by whether individuals improved or worsened after treatment. An analysis of average periodontal pocket depth before and after treatment showed that less than half (N = 12) the treated individuals improved post-treatment, while the rest stayed the same (N = 6) or worsened (N = 18). Lines indicate the proportion for a particular individual. The d-scores indicate the median line slope. **a** *Fusobacterium*, **b** *Prevotella*, **c** *Streptococcus*, **d** *Veillonella*. Note that the scale of the y-axis differs to highlight difference in individual responses to treatment.

In interpreting patient response to treatment, accounting for the personal microbiome of individual patients proved critical. This interpersonal variability also explains why we do not observe pre- and post-treatment clustering in PCoA space (Figure 1). While there are consistent changes associated with recovery from periodontal disease (e.g., a decrease in *Prevotella* abundance), the “healthy” amount of *Prevotella* differs on an individual basis. Moreover, the flora of some individuals changed contrary to the prevailing trends, notably in the *Fusobacterium* and *Prevotella*. *Streptococcus* remained steady or slightly increased in patients that improved, except two individuals who experienced dramatic declines post-treatment (Figure 3c). We also did not observe an expected increase in *Veillonella* in improving individuals post-treatment (Figure 3d).

**Figure 4:**
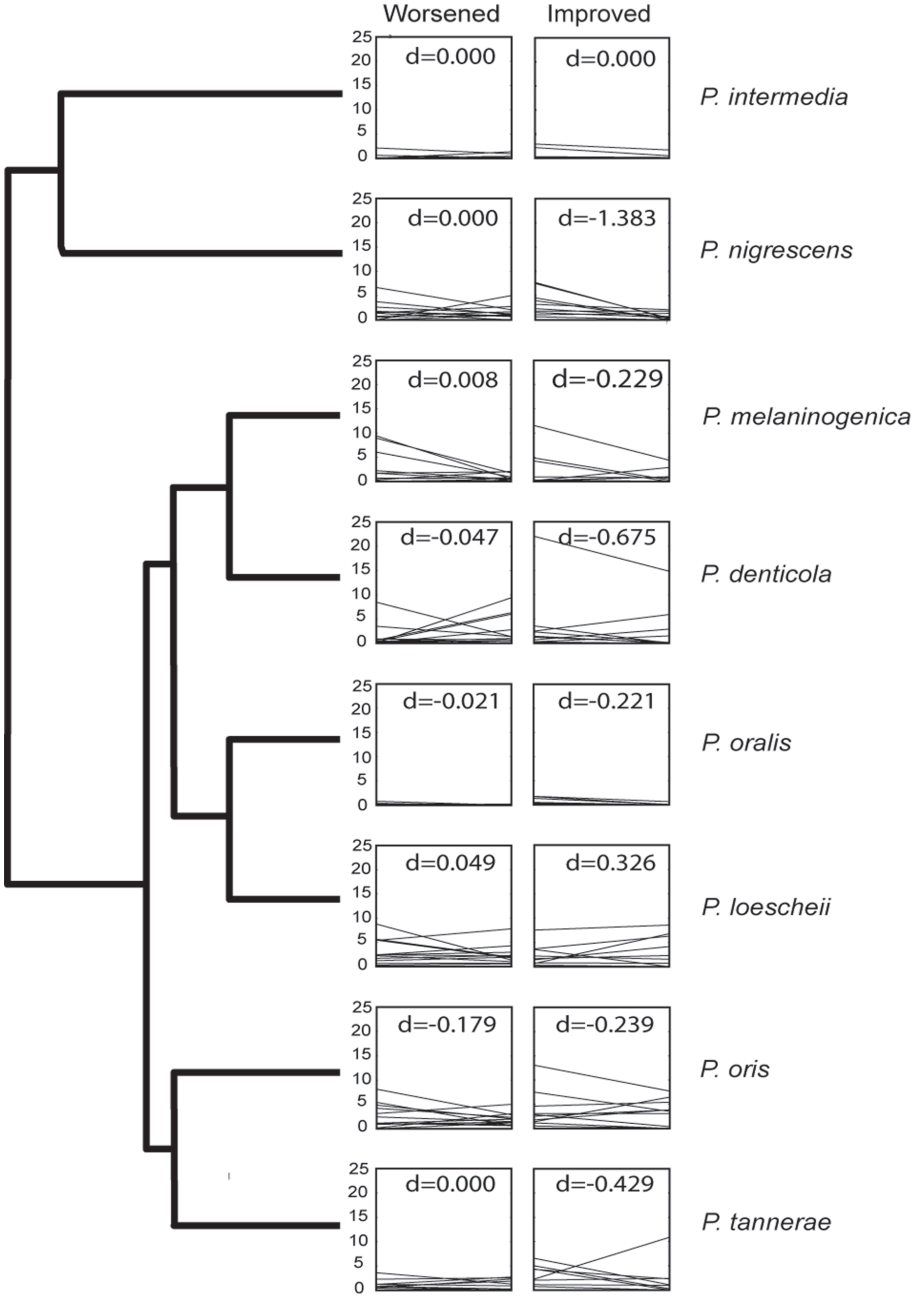
Representative cladogram of *Prevotella* species determined in this study (based on phylogenetic analysis shown in Figure S2) with plots of relative abundance of specific species divided into patients that improved and patients that worsened. The d-scores indicate the median line slope. In many cases, changes in relative proportions before and after treatment appeared to be species dependent.

**Figure 5:**
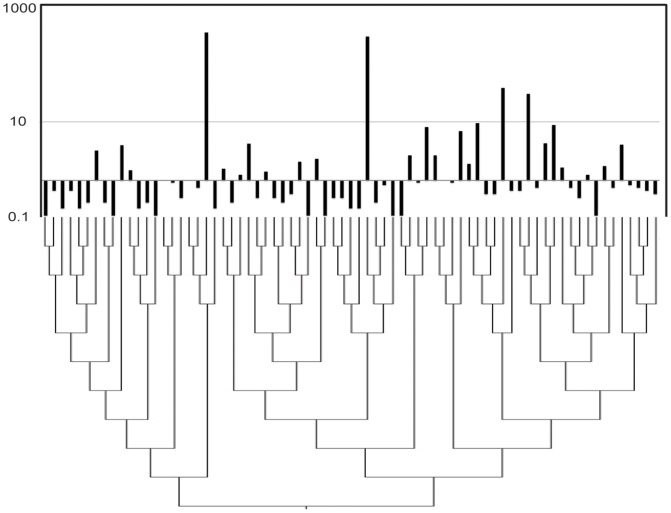
Cladogram of 73 different *Fusobacterium*-species (OTUs clustered at 97%) sequences along with a histogram showing the log OTU-count abundance of these same species. Most OTUs were sparse and the overall diversity within and among pockets was considerable.

Understanding the behavior of the biofilm response also appeared, at least in the case of *Prevotella*, to require more species-specific knowledge. Having successfully differentiated a number of oral *Prevotella* species (Figure S3), we found the abundance of *P. melaninogenica* and *P. loescheii* changed in opposite directions, while other *Prevotella* showed highly variable response post-treatment (Figure 4). A closer examination of *Fusobacterium* diversity also provided intriguing insight into periodontal biofilms. OTU clustering and phylogenetic analysis determined as many as 73 different species (Figure 5; Figure S4). Only four of these were abundant across all samples, and only two were found in every sample (Figure 5), supporting recent findings that the core human microbiome in unrelated individuals tends to be minimal at lower taxonomic levels [24]. These rarer species may increase the overall immune response and metabolic activity, but our data also suggest the presence of biofilm “cheaters” who contribute little to actual biofilm stability.

In the past, it was common to focus on the presence or absence of the bacteria that comprise the “red complex” (*Porphyromonas gingivalis*, *Tannerella forsythia* and *Treponema denticola*), which were implicated in disease [26]. However, it is clear from recent studies that culturing and emphasis on specific bacteria will not capture all the variability in the diseased periodontium [6], [9], [10]. This leads us to question the use of antibiotics in treatment of periodontal disease due to the variability of bacteria found in different diseased patients and the varied susceptibility of bacteria to different kinds of antibiotics.

Systemic antibiotic therapy is often used in periodontics to reduce or eradicate periodontopathic bacteria that are invading gingiva or are otherwise not reachable by topical antimicrobial treatment [25]. The selection of antibiotics is challenging because deep periodontal pockets can harbor several pathogens which exhibit diverse susceptibility to common antibiotics. Reference laboratories are available to identify periodontal pathogens and their antibiotic susceptibility, but most dentists institute antibiotic therapy empirically based on the best estimate of the most probable pathogen(s) and their usual antibiotic susceptibility pattern. Combination antibiotic therapy is frequently employed to cover a broader spectrum of pathogens. However, even though properly prescribed antibiotics can help provide resolution of severe periodontitis, the widespread use of antibiotics carries risks of inducing antibiotic resistance in important medical pathogens. It is expected that increased insights into the composition of the periodontal microbiome will lead to a better definition of patients who may, or may not, benefit from adjunctive antibiotic therapy.

Altogether, our results highlight the importance of understanding each patient's personal oral microbiome, a goal achievable by collecting and analyzing pre- and post-treatment samples. Furthermore, they lead us to believe that there is not a single composition that represents a healthy periodontal state and that recovery from periodontal disease appears to reflect a shift from a personalized disease state to a personalized healthy state. While there is consensus that particular communities should shift with response to disease, there may not be a “healthy amount” of these bacteria that is consistent across individuals. Further research with a larger patient sample size and more sampling over a longer time period will be necessary to confirm this hypothesis.

## Supporting Information

Figure S1 **TREND checklist for non-randomized trials.**
(PDF)

Figure S2 **Table describing the distribution of patients by disease classification.**
(PDF)

Figure S3 **Maximum likelihood tree of** ***Prevotella*** **-related small-subunit ribosomal RNA gene sequences.** The sequences highlighted in red were obtained in this study, while the rest include both cultured and uncultured sequences obtained from GenBank. To be included in the phylogenetic analysis, sequences identical to the representative OTU had to be found in at least three independent periodontal pocket samples. Sequences from cultured and uncultured organisms were also included in the alignments. Alignments were trimmed to ∼300 nucleotides and checked for accuracy and edited manually. Maximum-likelihood trees were created using RAxML HPC-BlackBox on CIPRES ([27]; http://www.phylo.org/). Black circles indicate bootstrap values of >70% while white circles indicate bootstrap values between 50 and 70%.
(PDF)

Figure S4 **Maximum likelihood tree of** ***Fusobacterium*** **-related small-subunit ribosomal RNA gene sequences.** The sequences highlighted in red were obtained in this study. The orange highlighted sequences were obtained from a study of bacteria in periradicular lesions by Saber *et al.* (2012) [27]. See Figure S3 for details on the phylogenetic methods.
(PDF)
