# Supplementary material for: The Personal Human Oral Microbiome Obscures the Effects of Treatment on Periodontal Disease
Source: PLoS One. 2014 Jan 29;9(1):e86708. doi: 10.1371/journal.pone.0086708 (PMC3906071; doi:10.1371/journal.pone.0086708)
Supplement: Figure S2 — Table describing the distribution of patients by disease classification. (PDF) [file pone.0086708.s002.pdf]

**Disease classification**	**Number of patients**
Healthy controls	4
Gingivitis	23
Mild-moderate periodontitis	12
Severe periodontitis	1
