# Supplementary figures and images for: The Personal Human Oral Microbiome Obscures the Effects of Treatment on Periodontal Disease
Source: PLoS One. 2014 Jan 29;9(1):e86708. doi: 10.1371/journal.pone.0086708 (PMC3906071; doi:10.1371/journal.pone.0086708)

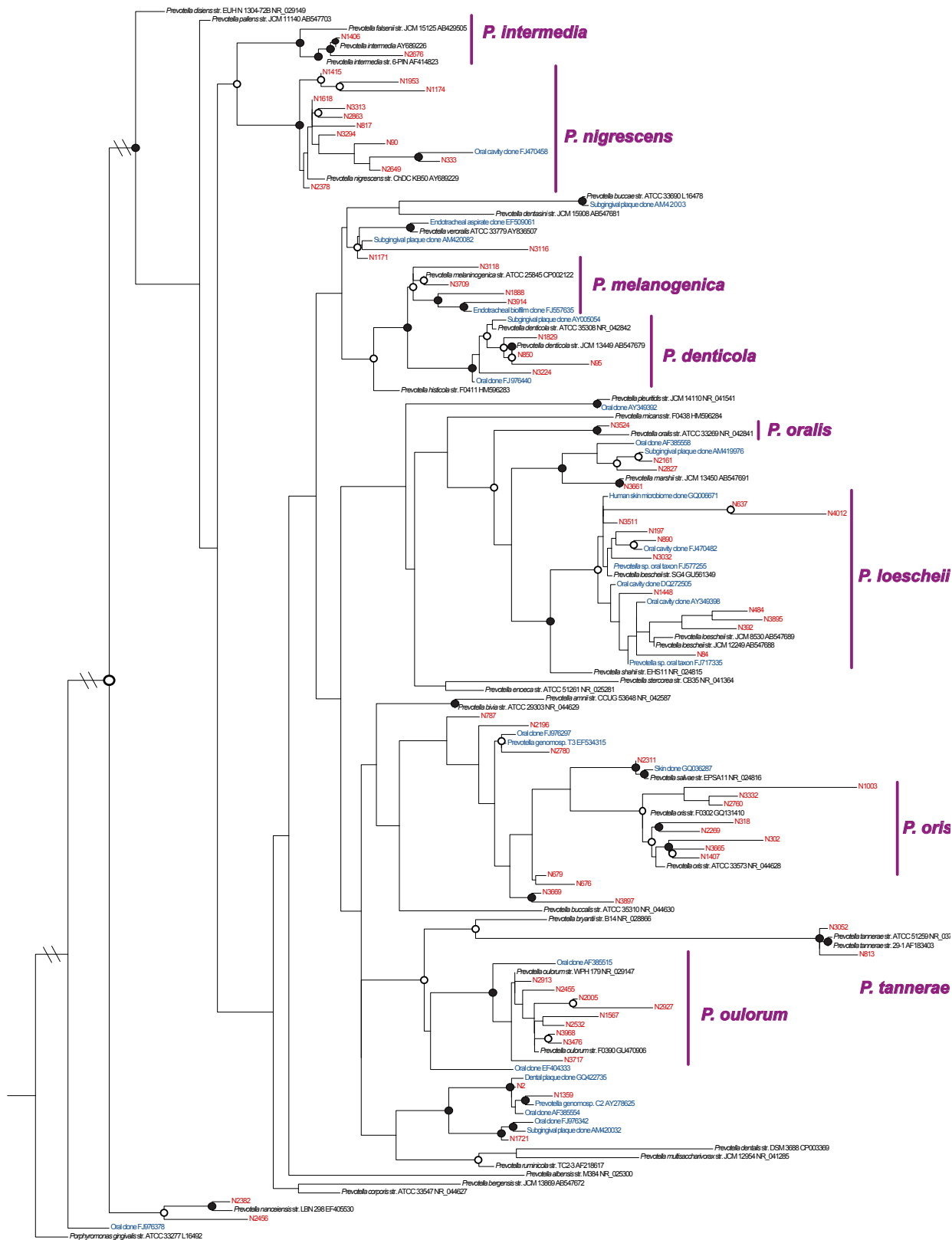

Supplement: Figure S3 — Maximum likelihood tree of Prevotella -related small-subunit ribosomal RNA gene sequences. The sequences highlighted in red were obtained in this study, while the rest include both cultured and uncultured sequences obtained from GenBank. To be included in the phylogenetic analysis, sequences identical to the representative OTU had to be found in at least three independent periodontal pocket samples. Sequences from cultured and uncultured organisms were also included in the alignments. Alignments were trimmed to ∼300 nucleotides and checked for accuracy and edited manually. Maximum-likelihood trees were created using RAxML HPC-BlackBox on CIPRES ([27]; http://www.phylo.org/). Black circles indicate bootstrap values of >70% while white circles indicate bootstrap values between 50 and 70%. (PDF) [file pone.0086708.s003.pdf]

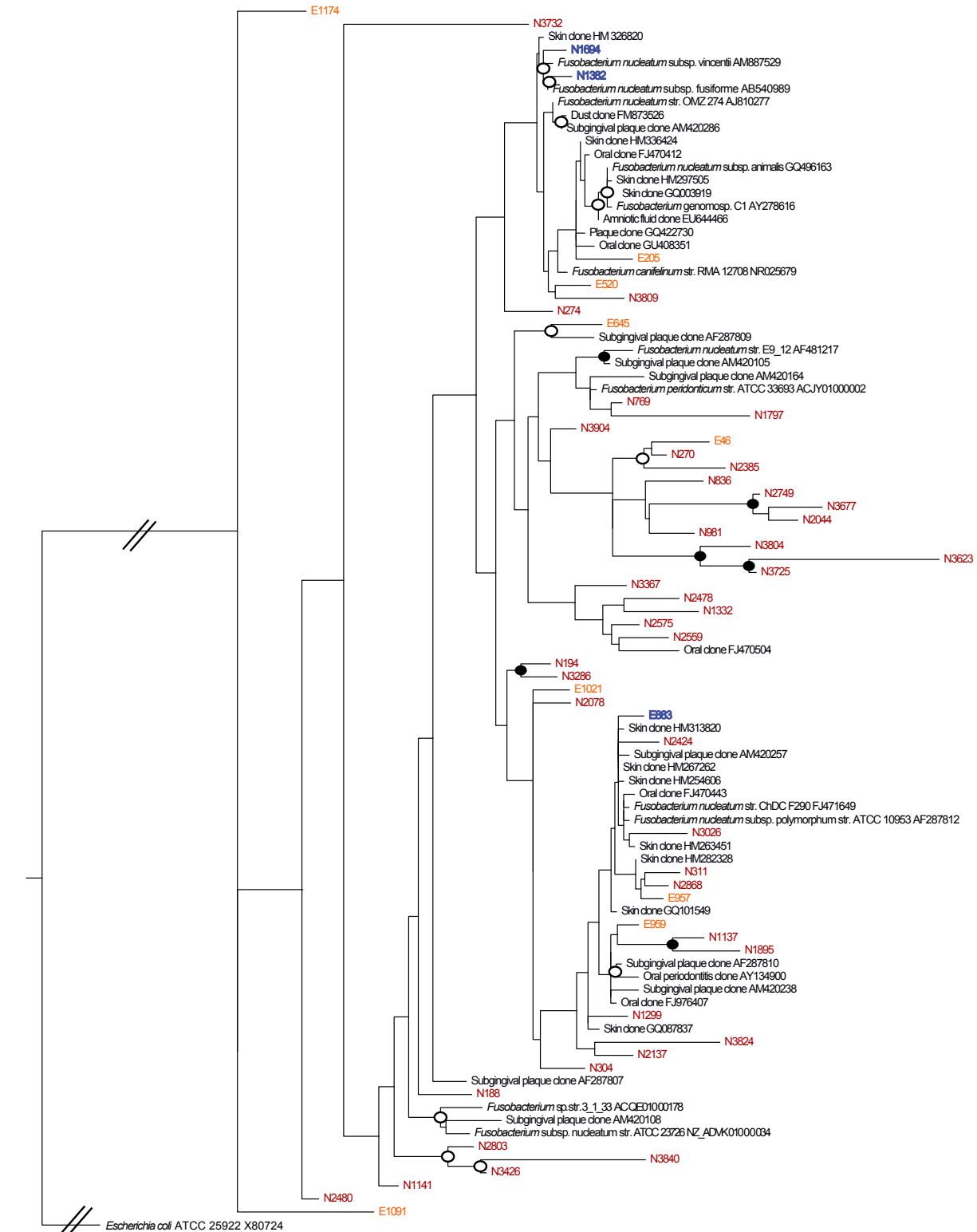

Supplement: Figure S4 — Maximum likelihood tree of Fusobacterium -related small-subunit ribosomal RNA gene sequences. The sequences highlighted in red were obtained in this study. The orange highlighted sequences were obtained from a study of bacteria in periradicular lesions by Saber et al. (2012) [27]. See Figure S3 for details on the phylogenetic methods. (PDF) [file pone.0086708.s004.pdf]
